# Plasmonic and magnetic ZnO-based nanocomposites for enhanced photocatalysis and ultrasensitive SERS detection of malachite green

**DOI:** 10.1038/s41598-026-51090-0

**Published:** 2026-05-19

**Authors:** H. Awad, KH. Hamdy, Y. Yasser, S. Magdy, N. Phlip, N. Fawzy, F. Ashraf, R. Mohammed

**Affiliations:** Physics Department, Faculty of Science, Ain Shams University, Cairo, Egypt

**Keywords:** Photocatalysis, Adsorption, ZnO NRs, Nanocomposites, SERS, Magnetite NPs, Chemistry, Environmental sciences, Materials science, Nanoscience and technology

## Abstract

Water pollution arising from persistent organic dyes constitutes a critical environmental and public health concern. In this work, ZnO nanorods (NRs), Fe_3_O_4_ nanoparticles (NPs), and their corresponding Ag@ZnO and Fe_3_O_4_@ZnO nanocomposites were successfully fabricated via a facile and cost-effective microwave-assisted hydrothermal approach. The photocatalytic activities of ZnO NRs, Fe_3_O_4_ NPs, and the as-prepared nanocomposites, including Ag@ZnO, were systematically evaluated for the degradation of malachite green (MG) dye. In addition, Ag@ZnO was further employed as an efficient active substrate for surface-enhanced Raman spectroscopy (SERS) sensing, demonstrating its dual functionality in both photocatalytic degradation and sensing applications. Structural, morphological, and optical properties were comprehensively characterized using X-ray diffraction (XRD), Fourier transform infrared (FTIR), Raman spectroscopy, diffuse reflectance spectroscopy (DRS), photoluminescence (PL), transmission electron microscopy (TEM), and dynamic light scattering (DLS) analysis. The XRD analysis confirmed the hexagonal wurtzite structure for both ZnO NRs and Ag@ZnO nanocomposites, in addition to the cubic structure for Fe_3_O_4_ NPs. The FTIR and Raman studies confirmed the formation of ZnO, Ag@ZnO, and Fe_3_O_4_ NPs by revealing their distinct characteristic vibrational bands. Zeta potential results display the negative surface charge of Fe_3_O_4_ NPs, indicating their strong adsorption affinity toward positively charged malachite green (MG) dye. The DRS and PL results reveal the effect of Ag NPs on improving the optical property of ZnO and reducing the (e–h) recombination rate. Photocatalytic experiments under visible-light irradiation showed that ZnO achieved 55% degradation of MG within 90 min, whereas complete degradation was achieved using Ag@ZnO and Fe_3_O_4_@ZnO nanocomposites, highlighting their superior photocatalytic performance. In addition, Ag@ZnO exhibited excellent SERS activity, enabling the detection of MG down to 2 ppm, attributed to strong localized surface plasmon resonance and interfacial charge-transfer effects. The combined photocatalytic efficiency, adsorption capability, and SERS sensitivity demonstrate that Ag@ZnO and Fe_3_O_4_@ZnO nanocomposites are promising multifunctional materials for wastewater treatment and trace-level pollutant detection.

## Introduction

Water scarcity has been a rising issue over the past decades, attracting the public’s concern. Wastewater pollution poses a significant threat to environmental and human health, with various industrial dyes being the primary contributors^1,2^. The disposal of dye-containing wastewater poses a significant environmental challenge due to its toxic nature. Among these pollutants, malachite green (MG) dye is particularly notorious due to its extensive use in the textile, aquaculture, and food industries, as well as its high toxicity and persistence in the environment. The presence of MG dye in wastewater is associated with severe adverse effects on human health, including mutagenic, carcinogenic, and teratogenic impacts^1,3,4^.

Addressing the challenge of MG dye removal from wastewater requires effective and sustainable treatment methods. Photocatalysis has emerged as a promising approach, leveraging the ability of photocatalysts to harness light energy to degrade organic pollutants^5–9^. Zinc oxide (ZnO) is a widely studied photocatalyst due to its favorable properties, such as high photosensitivity, chemical stability, and non-toxicity^10–14^. However, ZnO suffers from several drawbacks, including rapid electron–hole recombination and limited absorption in the visible light spectrum, which constrain its photocatalytic efficiency^15–17^.

To overcome these limitations, recent studies have explored surface modification and composite formation strategies^18–20^.

Ag-modified ZnO nanocomposites have shown enhanced photocatalytic performance for MG degradation^21,22^. For instance, electrospun ZnO/cellulose acetate/polypyrrole nanofibers surface-immobilized with Ag achieved 93.5% MG degradation within 1 h, compared to 63% for unmodified ZnO fibers^23^. Similarly, Ag-ZnO/polyaniline nanocomposites reached 98.58% MG degradation under visible light, highlighting the synergistic effect of Ag and conducting polymers in extending light absorption and improving charge separation^24–27^. In parallel, Fe_3_O_4_/ZnO composites have demonstrated substantial photocatalytic efficiency, with up to 99% Rhodamine B and 90% Evans blue degradation under UV light, attributed to heterojunction formation, enhanced charge separation, and increased surface area^28^. Further, ternary composites such as Fe_3_O_4_@ZnO@Bi_2_O_2.7_ achieved 98–100% degradation of various dyes under UV light, with improved electron–hole separation through heterojunction construction, while maintaining photocatalytic performance over multiple cycles^29^.

Despite the reported advances in ZnO-based photocatalysts, systematic comparative studies that directly evaluate plasmonic and magnetic modifications of ZnO prepared via the same synthesis route remain scarce, particularly for malachite green degradation. Moreover, most previous works focus on a single functionality, predominantly photocatalysis, without integrating adsorption behavior, recyclability, and sensing capability. In addition, conventional synthesis routes often require long reaction times, high energy input, and chemically intensive procedures, limiting their sustainability and scalability. In this context, the present study introduces a rapid microwave-assisted strategy for fabricating plasmonic Ag@ZnO and magnetic Fe_3_O_4_@ZnO nanocomposites, coupled with a green synthesis of Fe_3_O_4_ using flax (linseed) seed extract. This work uniquely combines visible-light-driven photocatalysis, adsorption, magnetic separation, and ultrasensitive SERS detection of MG, providing a comprehensive platform to elucidate how plasmonic and magnetic modifications distinctly influence ZnO performance.

In this work, we address this gap by synthesizing and comparatively investigating plasmonic Ag@ZnO and magnetic Fe_3_O_4_@ZnO nanocomposites via a microwave-assisted hydrothermal route, which enables rapid synthesis, low energy consumption, and facile scalability. Notably, Fe_3_O_4_ nanoparticles were synthesized using flax seed extract as a green, bio-mediated reducing and stabilizing agent, providing an environmentally friendly alternative to conventional chemical synthesis routes. ZnO nanorods, Fe_3_O_4_ nanoparticles, Ag@ZnO, and Fe_3_O_4_@ZnO were synthesized and comprehensively characterized using XRD, TEM, DLS, Raman spectroscopy, DRS, and PL analysis. Their photocatalytic performance for MG degradation was systematically evaluated under visible-light irradiation. In addition, the adsorption behavior of Fe₃O₄ and the SERS sensing capability of Ag@ZnO were also investigated. This study thus provides a detailed understanding of how plasmonic and magnetic modifications distinctly influence ZnO performance, offering insights into the design of multifunctional nanomaterials for efficient wastewater treatment.

## Experimental work

### Materials

Flaxseed, Ferrous Chloride (FeCl_2_), Ferric Chloride (FeCl_3_), Sodium Hydroxide (NaOH), Zinc Nitrate (Zn(NO_3_)_2_), Hexamethylenetetramine HMT (C_6_H_12_N_4_), Silver Nitrate (AgNO_3_), Polyvinylpyrrolidone PVP MW 10,000 (g/mol) (C_6_H_9_NO), methylene green (MG) dye, and Ethylene Glycol (C_2_H_6_O_2_).  All chemicals were purchased from Loba Company and used as received without further purification. Deionized water was used throughout all experiments.

### Preparation of ZnO nanorods

1 g of Zn(NO_3_)_2_ was dissolved in 25 mL of deionized water, while 0.5 g of HMT was separately dissolved in another 25 mL of deionized water. The two solutions were then mixed and stirred vigorously until a clear homogeneous solution was obtained. Subsequently, the resulting mixture was subjected to microwave irradiation at a power of 100 W. The microwave irradiation is turned on for 2 min, turned off for 30 s, and then repeated for 5 cycles. After heating, the white precipitate of ZnO is formed. Then, they were separated by filter paper and washed many times with deionized water. Finally, the precipitate is dried in an oven at 80°C for 6 h. The mechanism of the reaction is illustrated by the following Eqs. (1–4)^30,31^.

1 $$\left( {{\mathrm{CH}}_{2} } \right)_{6} {\mathrm{N}}_{4} + 6{\text{ H}}_{2} {\mathrm{O}} \to 6{\text{ H}}_{2} {\mathrm{CO}} + 4{\mathrm{NH}}_{3}$$

2 $${\mathrm{NH}}_{3} + {\mathrm{H}}_{2} {\mathrm{O}} \to {\mathrm{NH}}_{4}^{ + } + {\mathrm{OH}}^{ - }$$

3 $${\mathrm{Zn}}^{{2 + }} + {\text{ }}2\;{\mathrm{OH}} \to {\mathrm{Zn}}\left( {{\mathrm{OH}}} \right)_{2}$$

4 $${\mathrm{Zn}}\left( {{\mathrm{OH}}} \right)_{2} \xrightarrow{\Delta }{\mathrm{ZnO}} + {\mathrm{H}}_{2} {\mathrm{O}}$$

### Single-step synthesis of Ag@ZnO nanocomposite

1 g of Zn (NO_3_)_2_ dissolved in 25 ml of deionized water and 0.5 g of HMT in 15 ml were added under vigorous stirring, and 0.025 g of AgNO_3_ and 0.03 g of PVP dissolved in 10 ml of ethylene glycol were added to the mixed solution. A white–grey product is collected by filtration and washed with distilled water several times. Finally, the precipitate is dried in an oven at 80°C for 6 h.

### Synthesis of Fe_3_O_4_ magnetite nanocomposite^32^

A desired weight of flax seeds (provided from Western region farms, Egypt), washed with water, and dried, was suspended in deionized water and then boiled for 15 minutes. Fe_3_O_4_ NPs are synthesized by dropwise addition of freshly prepared flaxseed extract to a mixture of 0.2M FeCl_3_ and 0.1M FeCl_2_ (2:1 M ratio) and stirred until the color changes to dark brown. Then, 0.5 M NaOH was gradually added to the resulting suspension to adjust the pH to 11. After pH adjustment, the color of the suspension changed to black.  The resulting mixture was then further stirred using a magnetic stirrer for 2 h to ensure complete reaction and homogeneity. The solid magnetite particles were separated by filtration, washed several times until neutral, and then dried in an oven at 120°C for 1 h. The formation mechanism of Fe_3_O_4_ NPs can be represented by the following Eq. (5):

5 $${\mathrm{2Fe}}^{ + 3} + {\mathrm{Fe}}^{ + 2} + {8}\left( {{\mathrm{OH}}^{ - } \left[ {{\mathrm{FSE}}} \right]} \right) \to {\mathrm{2Fe}}\left( {{\mathrm{OH}}} \right)_{{3}} + {\mathrm{Fe}}\left( {{\mathrm{OH}}} \right)_{{2}} \to {\mathrm{Fe}}_{{3}} {\mathrm{O}}_{{4}} + {\mathrm{4H}}_{{2}} {\mathrm{O}}$$

### Preparation of Fe_3_O_4_@ZnO nanocomposite

ZnO and Fe_3_O_4_, with a molar ratio of 2:1, are added to 50 ml of deionized water. The suspension was further mixed in a magnetic stirrer for 1 h. Then heated in a microwave oven for 12 min with a 2-min duration cycle. The solid particles were separated by filter paper and washed and dried in an oven at 80°C for 9 h.

The microwave-assisted hydrothermal method offers several advantages over conventional hydrothermal routes, including reduced reaction time, rapid and uniform heating, and simplified processing. These features enhance reproducibility, lower energy consumption, and enable efficient nanocomposite synthesis within a significantly shorter time, highlighting its potential as a scalable and cost-effective approach.

### Characterization

The molecular vibrations and functional groups of the prepared materials have been measured using an FTIR spectroscopy (JASCO 7400). The XRD technique is used to examine the phase structure and crystallinity of the prepared material. An XRD Panalytical X’Pert PRO diffractometer with CuK (λ = 1.54059 Å) was used to examine the diffraction pattern. The crystallite size of the prepared samples is calculated from the XRD line-broadening measurement from the Debye–Scherrer equation Eq. (6)^33^ as follows:

6 $${\mathrm{Crystallite}}\;{\mathrm{Size}} = \frac{0.98\lambda }{{\beta \cos \theta }}$$

where K is the shape factor (0.9), λ is the Cu Kα wavelength (0.15406 nm), β is the corrected full width at half maximum (FWHM), and θ is the Bragg angle.

The size and shape of NPs were evaluated by a high-resolution transmission electron microscope, JEM 2100 HRT, at an accelerating voltage of 200 kV. The images were analyzed using ImageJ software to get the mean particle size and the standard deviation. The UV–Vis DRS of the samples was verified using a UV–Vis Shimadzu spectrophotometer (V-570) in the range of 200–1000 nm. The hydrodynamic size and the surface charge distribution were measured using the Zeta sizer Nano Series, a Malvern machine. A UV–Vis spectrophotometer (Ocean Optics) was used to measure the absorbance of the wastewater solution, which was then used to calculate the degradation percentage.

### Adsorption and photocatalytic degradation activities

Photocatalytic activities of the prepared samples are examined using MG in an aqueous solution with 10 ppm concentration as a model waste. 0.8 g L^−1^ of the prepared photocatalyst was also added, at pH 6. Using a magnetic stirrer, the solution was agitated in the dark for 30 min to achieve adsorption and desorption equilibrium. Then, the mixture was exposed to visible light using a 100 W visible light source. Continuous stirring was maintained throughout the experiment to ensure homogeneous mixing. At a regular interval of 15 min, 3 ml of the solution was withdrawn, filtered to remove the solid materials, and the dye concentration was measured using a UV–Vis spectrophotometer at 617 nm.

The adsorption efficiency and photocatalytic degradation efficiency were determined using the following Eqs. (7, 8):

7 $$Adsorption\, removal\, \mathrm{\%} = \frac{{C}_{0}-{C}_{t}}{{C}_{0}}\times 100$$

8 $$Degradation\, \mathrm{\%} = \frac{{C}_{0}-{C}_{t}}{{C}_{0}}\times 100$$

where C₀ is the initial waste concentration in the solution before adsorption or degradation, and C_t_ is the final waste concentration in the solution after adsorption and degradation.

Data analysis and plotting were performed using OriginPro (trial version, OriginLab Corporation, USA; https://www.originlab.com), while image processing and analysis were conducted using ImageJ (online version, National Institutes of Health, USA; https://imagej.nih.gov/ij/).

## Results and discussion

### XRD results

**Fig. 1:**
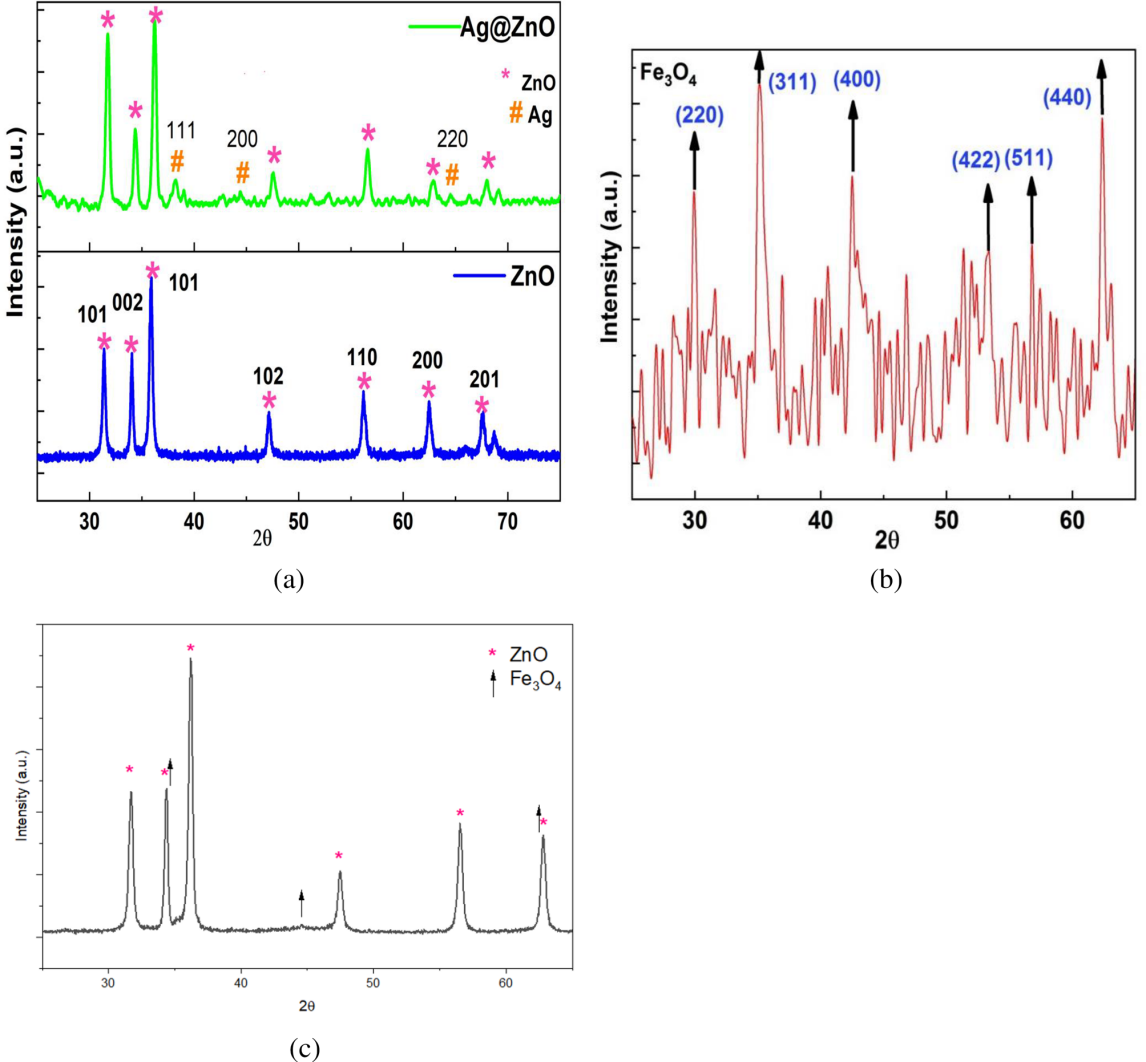
XRD pattern of (**a**) ZnO NRs, Ag@ZnO nanocomposite, (**b**) Fe_3_O_4_ NPs, and (**c**) Fe_3_O_4_@ZnO heterojunction.

The X-ray diffraction (XRD) analysis was employed to investigate the phase and crystal structure of the prepared ZnO NRs, Fe_3_O_4_ NPs,  as well as the Ag@ZnO and ZnO@Fe_3_O_4_ nanocomposites. The XRD patterns for ZnO NRs and Ag@ZnO nanocomposite are illustrated in Fig. 1. The diffraction peaks for both samples were analyzed and indexed according to ZnO hexagonal wurtzite structure according to JCPDS Card No. 96–900-4181 of ZnO (Wurtzite hexagonal phase)^34^.

For ZnO NRs, the characteristic diffraction peaks were observed at 2θ values of 31.8°, 34.5°, 36.3°, 47.6°, 56.7°, 63.0°, 68.1°, and 69.0°, corresponding respectively to the (100), (002), (101), (102), (110), (200), (201), and (004) planes, confirming the successful synthesis of crystalline ZnO^35^. The crystallite sizes of the synthesized materials were quantitatively estimated using the Scherrer’s equation based on the full width at half maximum (FWHM) of the main diffraction peaks, confirming their nanocrystalline nature^36^, and it was found to be 19.6 ± 3.56 nm, indicating a nanocrystalline structure.

The most intense peak at 36.3° corresponds to the (101) plane, indicating the preferred orientation, with a d-spacing of 2.47 Å.

Following Ag incorporation, the XRD pattern of the Ag@ZnO nanocomposite exhibited diffraction peaks analogous to those of pristine ZnO, with slight shifts toward lower 2θ values^21^.  The reflections at 31.3°, 34.0°, 35.9°, 47.2°, 56.2°, 62.5°, and 67.6° are indexed to the characteristic planes of hexagonal wurtzite ZnO, indicating structural preservation. The observed peak shift is attributed to lattice expansion induced by interfacial strain and defect generation arising from the interaction between Ag nanoparticles and the ZnO framework. The deposition of Ag nanoparticles on the ZnO surface can generate compressive or tensile stress and modify the local Zn–O bonding environment, leading to an increase in the interplanar spacing. In addition, the possible partial incorporation of Ag species with a larger ionic radius than Zn^2+^ can further contribute to lattice distortion. According to Bragg’s law, this increase in lattice spacing results in a shift of the diffraction peaks toward smaller 2θ values, while the overall crystal structure of ZnO remains unchanged^37^.

Additionally, new diffraction peaks at 38.1°, 44.3°, and 64.4° were observed in the XRD pattern of Ag@ZnO nanocomposite, which are attributed to the (111), (200), and (220) planes of cubic metallic silver structure (JCPDS no. 04–0783)^38^, respectively. These findings provide clear evidence for the successful incorporation of Ag nanoparticles onto the ZnO nanorod surface ^39^.

The calculated crystallite size was 19.7 ± 3.59 nm, is consistent with that of pristine ZnO, suggesting that Ag incorporation primarily takes place via surface deposition rather than Lattice substitution. The presence of metallic Ag is known to enhance photocatalytic performance by forming Schottky barriers at the Ag@ZnO interface, which trap photoexcited electrons and suppress recombination, as well as by contributing plasmonic resonance that extends the optical response into the visible region.

The absence of extra diffraction peaks in all XRD patterns indicates high phase purity of the prepared samples, further supporting the successful formation of the nanocomposites without secondary phases.

On the other hand, Fig. 1(b) illustrates the XRD pattern of Fe_3_O_4_ nanoparticles exhibited diffraction peaks at 2θ ≈ 30.1°, 35.4°, 43.0°, 53.4°, 57.0°, and 62.6°, corresponding to the (220), (311), (400), (422), (511), and (440) planes of cubic spinel magnetite (JCPDS no. 19–0629)^40^. The absence of any additional diffraction peaks indicates the high phase purity of the synthesized Fe_3_O_4_ nanoparticles. Furthermore, the average crystallite size was estimated to be approximately 18.45 nm, confirming the formation of nanocrystalline magnetite.

For the Fe_3_O_4_@ZnO nanocomposite, the XRD pattern revealed that the characteristic diffraction peaks of hexagonal wurtzite ZnO remained dominant, indicating that the crystal structure of ZnO is well preserved after coupling with Fe_3_O_4_. The diffraction peak of Fe_3_O_4_ corresponding to the (311) plane, located at 2θ ≈ 35.4°, is overlapped with the ZnO (002) peak, while the Fe_3_O_4_ (440) reflection, around 2θ ≈ 62.9°, is embedded within the ZnO (200) peak, making these magnetite peaks less distinguishable in the composite pattern. In addition, the Fe_3_O_4_ peak corresponding to the (400) plane at 2θ ≈ 43.0° is still observed, confirming the presence of the cubic spinel magnetite phase (JCPDS no. 19–0629). The relatively low intensity and partial masking of Fe_3_O_4_ peaks can be attributed to the lower content and lower degree of crystallinity of Fe_3_O_4_ within the composite, as well as its high dispersion on the ZnO nanorods. The absence of additional impurity peaks confirms the successful formation of the Fe_3_O_4_@ZnO heterostructure without secondary phases.

The preservation of ZnO peak positions, without noticeable shifts or structural distortion, indicates that Fe_3_O_4_ nanoparticles are primarily anchored on the surface of ZnO rather than incorporated into its crystal lattice. This structural configuration is favorable for forming a magnetic–semiconductor heterojunction, which can enhance charge separation efficiency during photocatalytic processes.

### FTIR results

**Fig. 2:**
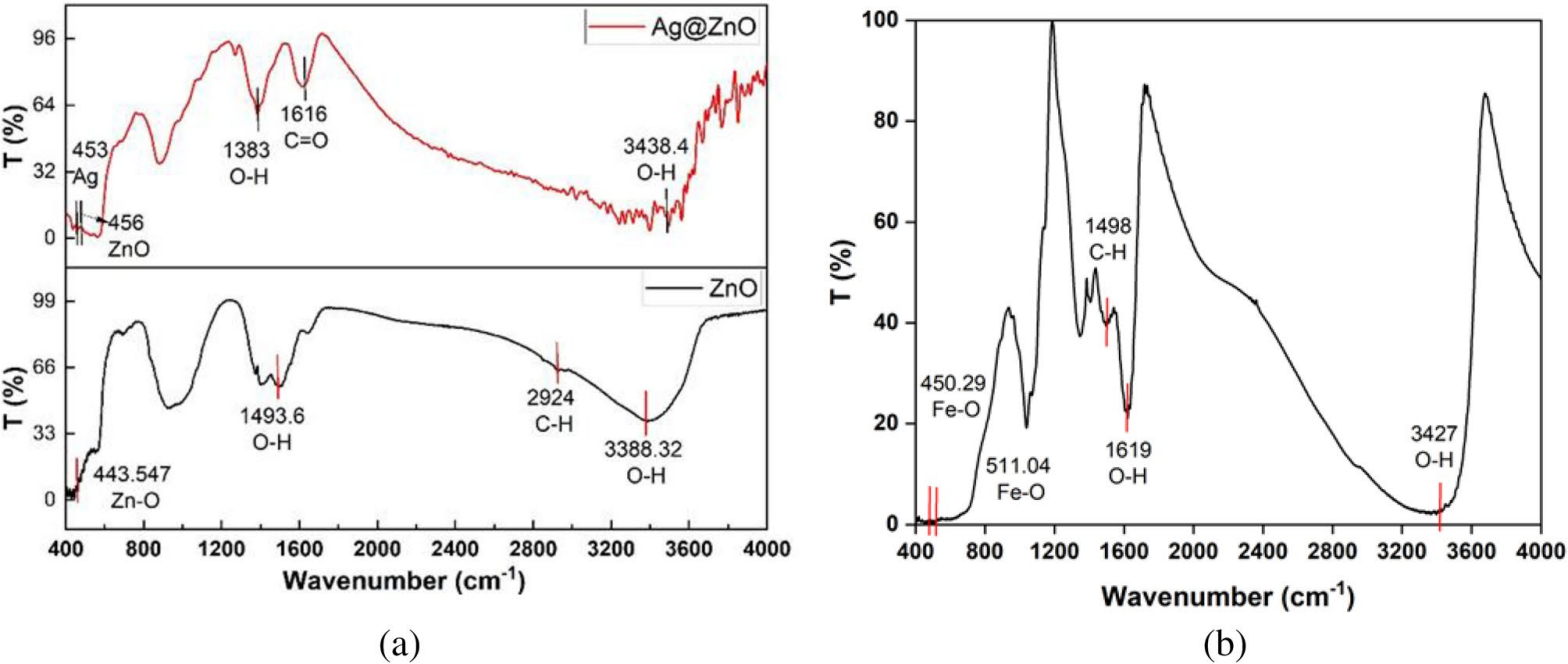
FTIR spectra for (**a**) ZnO NRs and Ag@ZnO nanocomposite, (**b**) Fe_3_O_4_ NPs.

The FTIR analysis is used to study the bond structure and the functional groups of the prepared nanomaterials. Fig. 2(a, b) illustrates the FTIR spectra of the ZnO NRs, Fe_3_O_4_ NPs_,_ and Ag@ZnO nanocomposite, in the range 400-4000 cm^-1^.

For the ZnO spectrum, the transmittance peak at 443.547 cm^−1^ is due to the Zn–O bond, which confirms the formation of the ZnO bond. The presence of water molecules adsorbed on the surface results in the appearance of two bands at 1493 cm^-1^ and 3388. cm^−1^, which are associated with O–H bending and stretching bands, respectively. The weak band at 2924 cm^−1^ is attributed to C–H bending vibrations arising from residual hexamine molecules adsorbed on the ZnO surface, which can be diminished upon calcination^41^.

For the Ag@ZnO nanocomposite, the main peak of ZnO present at 443.547 cm⁻^1^ is shifted to 456 cm^−1^ due to the presence of Ag; also, a new peak of Ag appears at 453 cm^−1^, which confirms the formation of Ag@ZnO nanocomposite. Furthermore, a peak at 1616 cm⁻^1^ corresponds to C=O stretching vibrations, possibly from polyvinylpyrrolidone (PVP). O–H bending and stretching bands appear at 1383 cm^−1^ and 3438.4 cm^−1^, respectively^42^.

The FTIR spectra of the Fe_3_O_4_ NPs exhibited vibrational bands at 450 and 511 cm^−1^ assigned to the IR active mode corresponding to the vibration of the Fe–O group, which confirms the formation of Fe_3_O_4_ NPs^43^. The appearance of two peaks for the Fe–O bond is attributed to the difference in the bond length of the atoms in two different atomic sites. Furthermore, the peak at 1498 cm^−1^ may be related to bending vibrations of C–H bonds from organic compounds present in flaxseed. The broad band at 1619 cm^−1^ and 3427 cm^−1^  which correspond to O–H bending and stretching vibrations, respectively^44^.

### Raman results

**Fig. 3:**
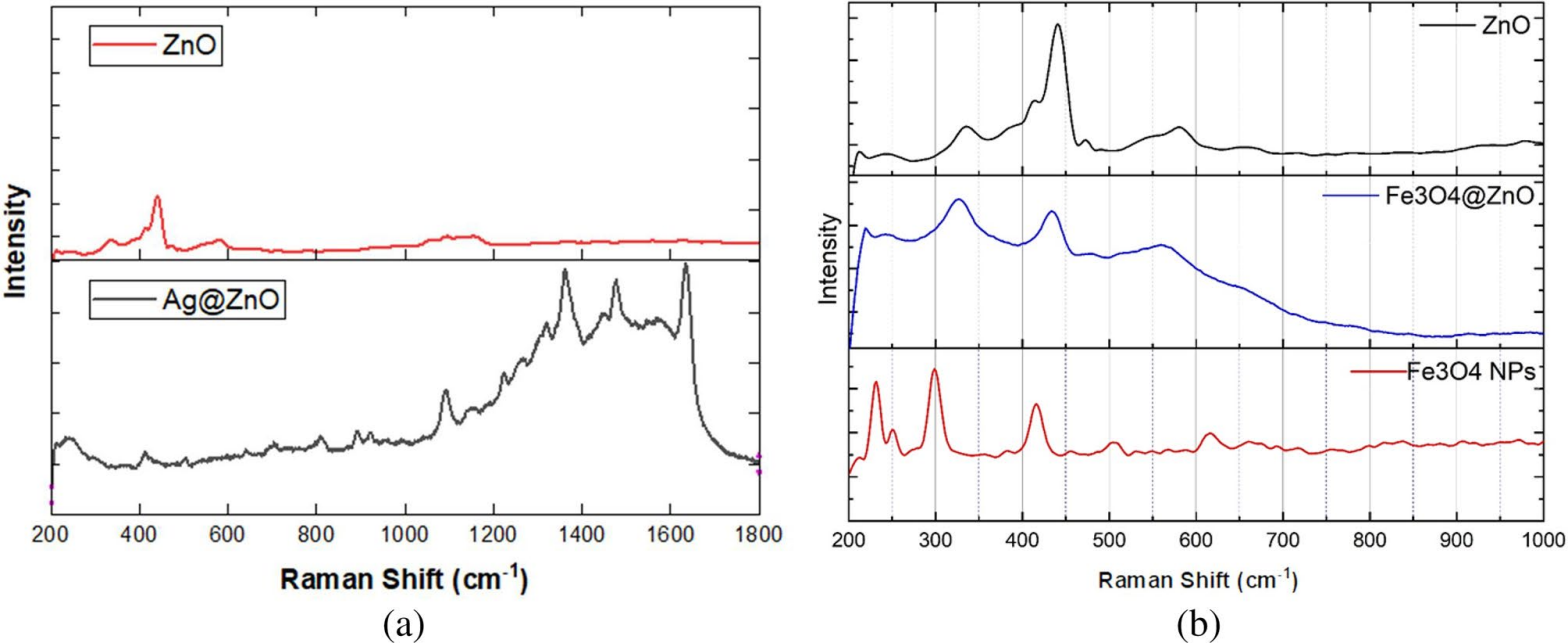
Raman spectra of (**a**) ZnO NRs and Ag@ZnO nanocomposite, (**b**) ZnO NRs, Fe_3_O_4_@ZnO nanocomposite, and Fe_3_O_4_ NPs.

The Raman spectra are carried out to study the structural and chemical bonding characteristics of ZnO NRs, Fe_3_O_4_ NPs, as well as the Ag@ZnO and Fe_3_O_4_@ZnO nanocomposites at their interfaces, as shown in (Fig. 3). In the spectrum of ZnO NRs, characteristic peaks associated with the wurtzite crystal structure of zinc oxide are typically observed, which shows the successful formation of ZnO NRs. These peaks include the prominent E_2_(high) mode at around 442 cm^−1^, attributed to the vibration of oxygen atoms in the Zn–O bond, and the A_1_(LO) mode at approximately 335 cm⁻^1^ and 580 cm⁻^1^, indicative of the longitudinal optical phonon mode^45^.

Upon the introduction of silver (Ag) nanoparticles onto the surface of ZnO, changes in the Raman spectrum occur, reflecting alterations in chemical bonding. The presence of Ag nanoparticles may lead to shifts of the ZnO peaks, an increase in the peak intensity due to surface plasmon resonance SPR effect, as well as the appearance of additional peaks corresponding to Ag-related vibrational modes^39^. The main ZnO NRs peak, which appears at 442 cm^−1^, is slightly red-shifted to 412.5 cm^−1^. For instance, Ag–O related peaks of the A1 mode at around 240 cm^−1^ are observed in the Raman spectrum of Ag@ZnO nanocomposite, indicating the presence of silver species^46^.

The intensity of the five peaks located at 643 cm^−1^, 705 cm^−1^, 1360 cm^−1^, 1479 cm^−1^, and 1635 cm^−1^ is significantly enhanced due to the presence of Ag in the Ag@ZnO nanocomposite structure^47^.

For the Fe_3_O_4_ Raman spectrum, the appearance of the main characteristic peaks of Fe_3_O_4_ at 300.3 cm^−1^, 504 cm^−1^, and 614 cm^−1^ confirms its successful formation. For the Fe_3_O_4_@ZnO spectrum, the main peak of ZnO merged with the Fe_3_O_4_ peaks and appears at 327 cm^−1^ and 435 cm^−1^^48,49^.

### TEM results

**Fig. 4:**
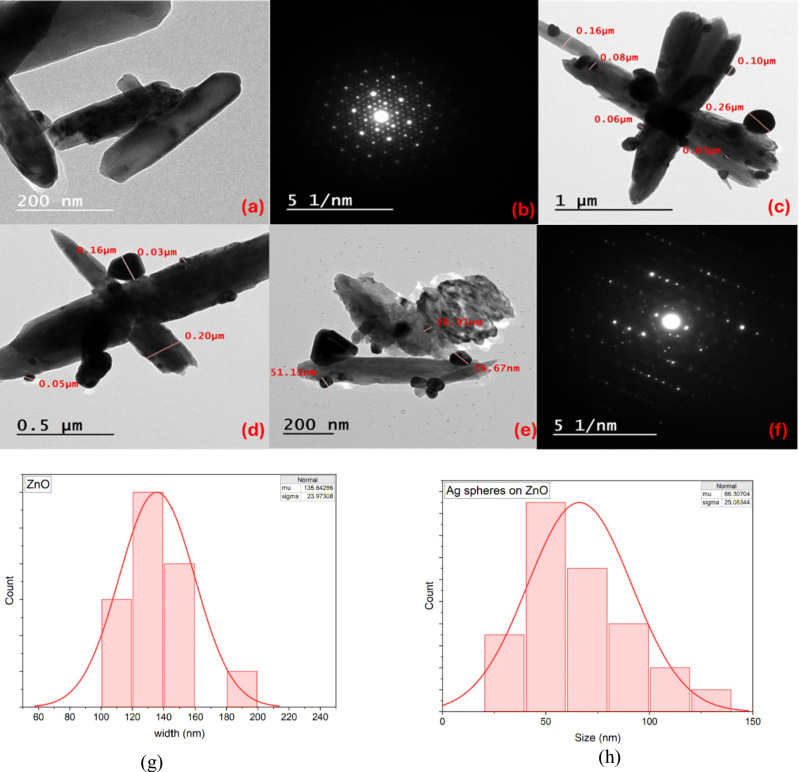
(**a**) TEM images of ZnO NRs, (**b**) the selected area electron diffraction pattern of ZnO NRs, (**c**, **d**, **e**) TEM images of Ag@ZnO nanocomposite, (**f**) the selected area electron diffraction pattern of Ag@ZnO nanocomposites, (**g**) the size histogram of Ag@ZnO nanocomposite and (**h**) Ag nanoparticles.

**Fig. 5:**
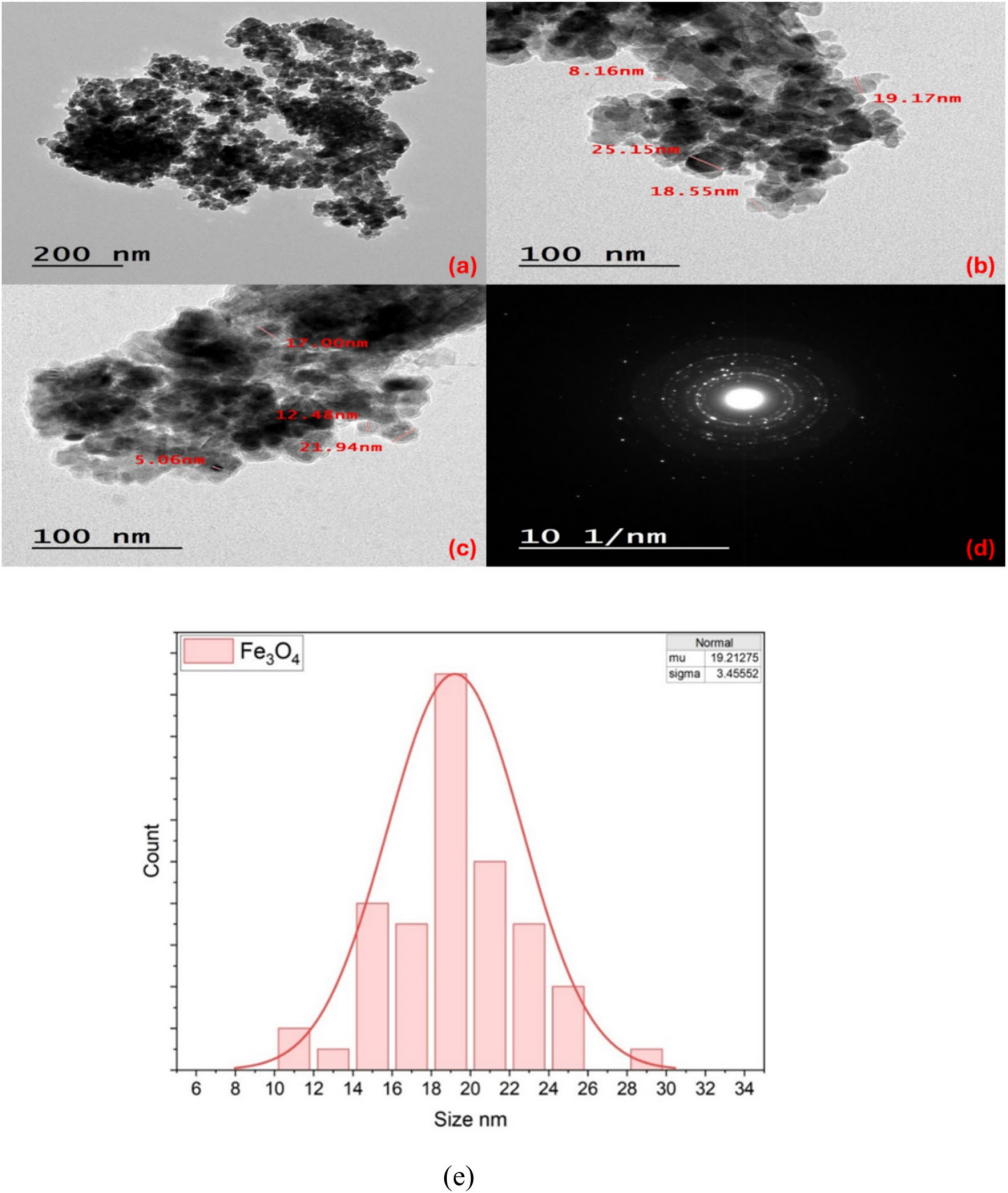
(**a**, **b**, **c**) TEM images of Fe_3_O_4_ NPs, (**d**) the selected area electron diffraction pattern of Fe_3_O_4_ NPs, (**e**) the size histogram of Fe_3_O_4_ NPs.

The TEM imaging technique was employed to examine the nanostructureal morphology of the prepared nanomaterials. Figure 4(a) shows that ZnO exhibits a well-defined nanorod-like morphology. TEM images of the Ag@ZnO nanocomposite (Fig. 4(c–e)) clearly reveal the decoration of Ag nanoparticles on the surface of the ZnO NRs, confirming the successful formation of the nanocomposite. The size distribution histograms in Fig. 4(g, h) indicate that the ZnO NRs have an average diameter of 135 ± 23 nm, while their average length is 1.71 ± 0.048 μm (length distribution not shown). In addition, the Ag nanoparticles display an average size of 66.3 ± 25 nm. Quantitative size and length measurements were performed using ImageJ software by analyzing approximately 80 individual nanostructures from representative TEM micrographs, and the reported values correspond to the calculated mean ± standard deviation. The ZnO nanorods exhibit a relatively narrow size distribution and good uniformity, and their aspect ratio confirms the formation of well-defined rod-like structures. For the Ag@ZnO nanocomposite, Ag nanoparticles are uniformly dispersed on the ZnO surface without significant agglomeration. Similarly, the Fe_3_O_4_ nanoparticles show a consistent nanoscale size distribution and are homogeneously dispersed. These statistical results confirm the structural uniformity and effective formation of the nanocomposites. The selected area electron diffraction (SAED) patterns shown in Fig. 5(b, f) provide further insight into the crystalline nature of the samples. The SAED pattern of pure ZnO NRs exhibits a series of well-defined bright diffraction rings, which can be indexed to the hexagonal wurtzite structure of ZnO, corresponding to the (100), (002), (101), (102), and (110) planes. This confirms the polycrystalline nature and high crystallinity of the ZnO nanorods.

In contrast, the SAED pattern of the Ag@ZnO nanocomposite displays, a slight variation in the ZnO diffraction rings indicates that the introduction of Ag nanoparticles slightly modifies the crystallinity of ZnO, possibly due to lattice strain or surface interaction between Ag and ZnO.

The TEM micrographs in Fig. 5 (a, b, c) show that the Fe_3_O_4_ sample is made up of quasi-spherical nanoparticles. Measured particle diameters on the images are about 19 ± 3.5 nm, indicating a polydisperse sample of nanoscale crystallites. At higher magnification, the particles appear faceted. The SAED pattern (Fig. 5 d) displays a set of concentric diffraction rings rather than discrete spots, which is characteristic of a polycrystalline ensemble of small Fe_3_O_4_ crystallites. This morphology and size distribution are consistent with the XRD data.

### DLS results

**Fig. 6:**
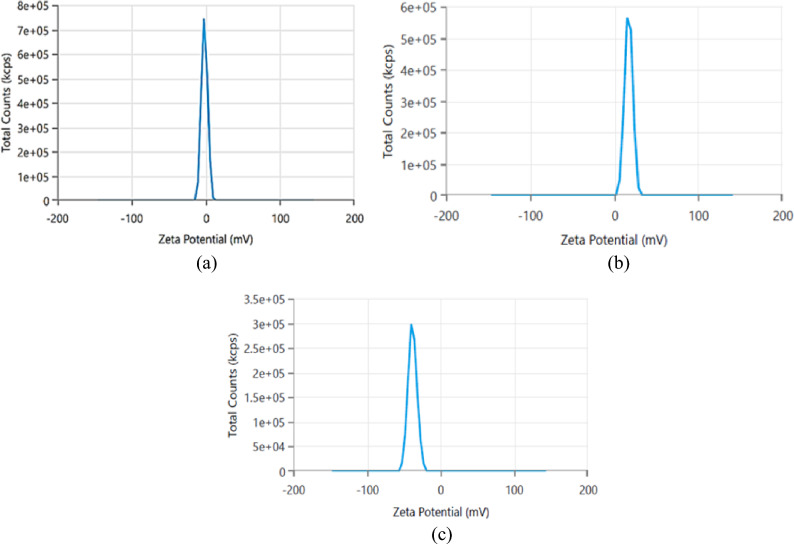
Zeta potential of (**a**) ZnO NRs, (**b**) Ag@ZnO Nanocomposite, and (**c**) Fe_3_O_4_ NPs.

DLS measurements provide valuable insights into the hydrodynamic size, surface charge, and stability of prepared materials and help in understanding the interaction between nanoparticles. The Zeta potentials of both ZnO NRs and Ag@ZnO nanocomposite are investigated and illustrated in Fig. 6 (a). In this study, pristine ZnO NRs exhibited a slightly negative zeta potential of approximately − 1.875 mV, which is consistent with the typical behavior of ZnO arising from surface hydroxyl groups and oxygen vacancies, as also supported by the FTIR results in Fig. 2 (a). Furturemore , the negative zeta potential is expected to enhance the adsorption of cationic MG dye, thereby improving contaminant removal from aqueous solutions^50^.

The reduction of silver (Ag) ions and their incorporation into the ZnO resulted in a significant shift in the zeta potential of the composite material. The Ag@ZnO nanocomposite exhibited a positive zeta potential of approximately +19 mV Fig. 6 (b), indicating a reversal in surface charge compared to pristine ZnO. This observation suggests a modification of the surface chemistry and stability upon the introduction of Ag NPs. These results can affect the adsorption ability of ZnO NRs and Ag@ZnO nanocomposite, as we will discuss later.

Moreover, the Zeta potential of Fe_3_O_4_ NPs was found to be approximately −38 mV Fig. 6 (c). This value is due to the negative surface charge typically exhibited by iron oxide nanoparticles due to the presence of surface hydroxyl groups. The high Fe_3_O_4_ NPs’ negatively charged surface will help in increasing their adsorption activity of the positively charged MG dye^51^.

### DRS results

**Fig. 7:**
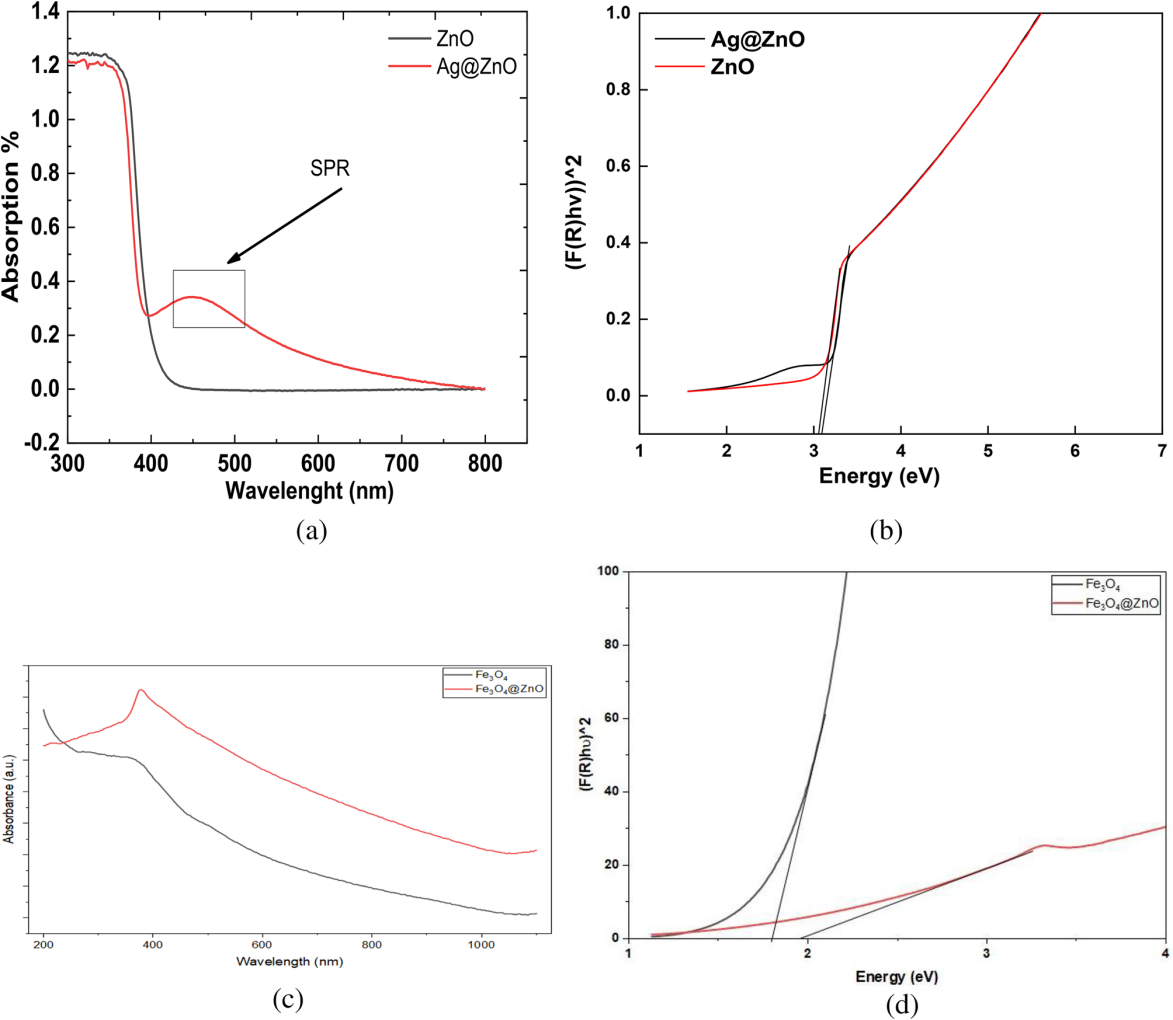
(**a**) DRS absorption spectrum for ZnO NRs and Ag@ZnO, (**b**) Tauc plots used to estimate the optical band gap energies of ZnO NRs, and Ag@ZnO nanocomposite, (**c**) absorption spectrum for Fe_3_O_4_ NPs, and Fe_3_O_4_@ZnO nanocomposite (**d**) Tauc plots used to estimate the optical band gap energies of Fe_3_O_4_ NPs, and Fe_3_O_4_@ZnO nanocomposite.

DRS spectra of ZnO nanoparticles and Ag@ZnO nanocomposites are shown in Fig. 7. The pure ZnO sample (black curve) exhibits a strong absorption edge around 370 nm, which corresponds to its intrinsic band gap transition, characteristic of ZnO’s wide band gap semiconductor nature. In contrast, the Ag@ZnO nanocomposite (red curve) shows an additional broad absorption band in the visible region, centered around 450–500 nm. This feature is attributed to the surface plasmon resonance (SPR) of silver nanoparticles embedded in the ZnO matrix. The presence of the SPR peak confirms the successful incorporation of Ag nanoparticles, which enhance visible light absorption. This extended absorption into the visible region suggests improved light-harvesting ability, making Ag@ZnO more effective for photocatalytic and optoelectronic applications compared to pure ZnO.

From Fig. 7,   the presence of surface plasmon resonance (SPR) can be observed, which arises in metallic nanoparticles due to the collective oscillation of conduction electrons induced by the electromagnetic field of the incident light.  In the case of Ag@ZnO, a distinct SPR band appears at 449.886 nm, attributed to the incorporation of Ag nanoparticles^52^.

The optical absorption properties of the synthesized ZnO NRs, Ag@ZnO nanocomposite, and Fe_3_O_4_ NPs were investigated using UV–Vis diffuse reflectance spectroscopy (DRS), and the corresponding spectra are presented in Fig. 7 (a, c). The optical band gap (E_g_) of the materials was estimated using the Kubelka–Munk function F(R) (Eq. (9, 10))^53^:

8 $${\mathrm{F}}\left( {\mathrm{R}} \right) = {{\left( {{1} - {\mathrm{R}}} \right)^{{2}} } \mathord{\left/ {\vphantom {{\left( {{1} - {\mathrm{R}}} \right)^{{2}} } {\left( {{\mathrm{2R}}} \right)}}} \right. \kern-0pt} {\left( {{\mathrm{2R}}} \right)}}$$

And the Tauc relation:

9 $$\left[ {F\left( {\mathrm{R}} \right) \cdot {\mathrm{h}}\nu } \right]^{{\mathrm{n}}} = {\mathrm{A}}\left( {{\mathrm{h}}\nu - {\mathrm{Eg}}} \right)$$

where hν is the photon energy, A is a constant, and n is an exponent that depends on the transition type (n = 1/2 for direct allowed transitions, as in ZnO).

The band gap energy values were determined by extrapolating the linear region of the Tauc plot, Fig. 7 (b, d). For pristine ZnO, a sharp absorption edge was observed near 400 nm, corresponding to a calculated band gap of 3.08 eV, in good agreement with reported values for ZnO semiconductors. Upon incorporation of Ag, the Ag@ZnO nanocomposite exhibited a noticeable red-shift with an additional broad absorption tail into the visible region (414 nm) corresponding to a calculated band gap of 2.99 eV. Importantly, a distinct plasmonic absorption peak appeared around 449.9 nm, which is attributed to the surface plasmon resonance (SPR) of metallic Ag nanoparticles. The presence of this SPR peak confirms the successful incorporation of Ag and its strong interaction with ZnO. The optical band gap of the composite was slightly reduced to 3.04 eV, indicating that Ag loading effectively narrows the band gap while simultaneously enhancing visible-light absorption. This synergistic effect improves charge carrier separation and broadens the spectral response, which is favorable for photocatalytic activity^54^.

The optical absorption properties of Fe_3_O_4_ NPs and Fe_3_O_4_@ZnO nanocomposite were investigated using diffuse reflectance spectroscopy, and the corresponding Tauc plots. The pristine Fe_3_O_4_ NPs sample exhibits a gradual absorption edge with no sharp transition, indicating its narrow band gap of about 1.82 eV^55^. In contrast, the Fe_3_O_4_@ZnO nanocomposite shows a more defined linear region in the higher energy range, characteristic of a direct band gap semiconductor due to the presence of ZnO. The extrapolation of the linear portion of the curve suggests an apparent band gap in the UV region, while the extended absorption into the visible region is attributed to the contribution of Fe_3_O_4_. This red-shifted absorption behavior indicates improved visible-light harvesting in the composite, which can be ascribed to the interfacial interaction between Fe_3_O_4_ and ZnO. Such coupling is expected to enhance charge separation efficiency and broaden the photoresponse range, making the Fe_3_O_4_@ZnO system more suitable for photocatalytic applications under solar irradiation.

Overall, the DRS results confirm that Ag incorporation not only narrows ZnO’s band gap but also introduces a plasmonic response in the visible region, while Fe_3_O_4_ contributes broad absorption across the UV–visible range. These combined optical properties make ZnO–Ag and Fe_3_O_4_ promising candidates for efficient photocatalytic applications.

### PL results

PL spectroscopy is a widely used technique for evaluating the separation efficiency of photogenerated charge carriers in semiconductors. PL emission originates from the radioactive recombination of photogenerated electrons and holes, and a low PL intensity is generally indicative of a low recombination rate.

**Fig. 8:**
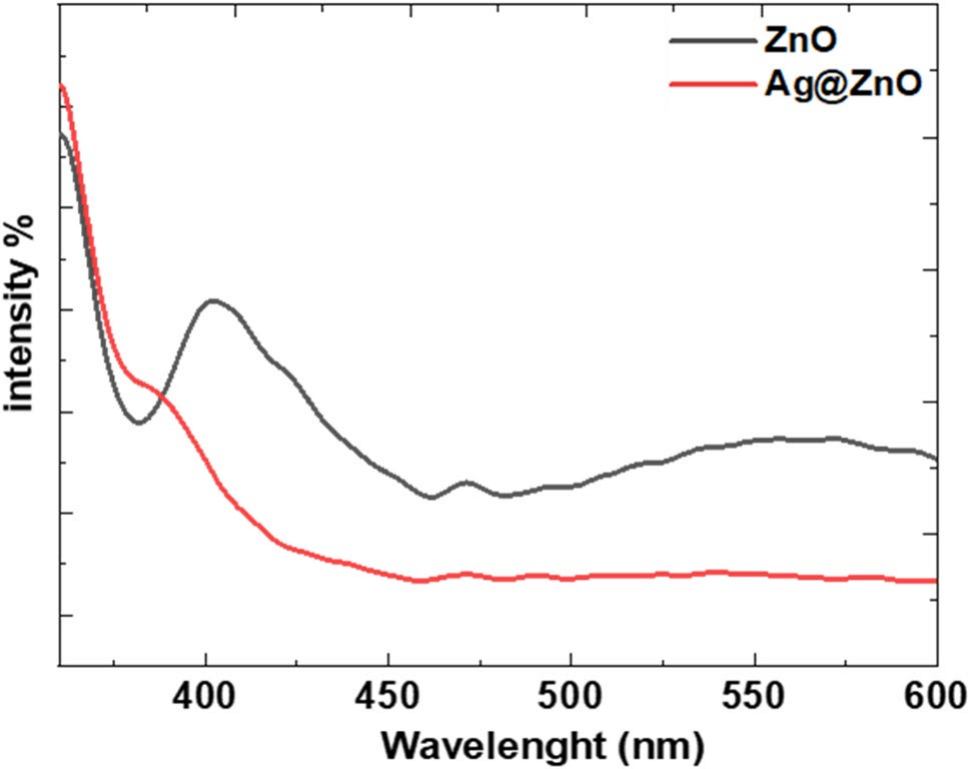
The room-temperature PL spectra of the ZnO NRs, and Ag@ZnO nanocomposite.

Figure 8 shows the room-temperature PL spectra of the ZnO and Ag-ZnO nanocomposite measured with an excitation wavelength of 350 nm.

The UV emission could be assigned to the near-band gap emission of the wide ZnO band gap, while the other two bands in the visible region could be attributed to bound excitons and defect states located at the surface of the nanostructured ZnO, respectively. The emission intensity of the Ag@ZnO PL spectrum has decreased with the addition of the Ag NPs. This suggests a decreased electron–hole recombination rate, and hence, increases in the lifetime of the photogenerated charge carriers^56^. In general, the efficient charge separation and inhibition of electron–hole recombination because of the Ag NPs enhances the photocatalytic activity of the ZnO nanostructures. Moreover, the band at 560 nm in the ZnO PL spectrum is not present in the Ag@ZnO PL spectrum, indicating that the deposition of Ag NPs onto the ZnO defect sites reduces the number of surface defects in the Ag@ZnO nanostructures^57^.

### Adsorption activity of Fe_3_O_4_

**Fig. 9:**
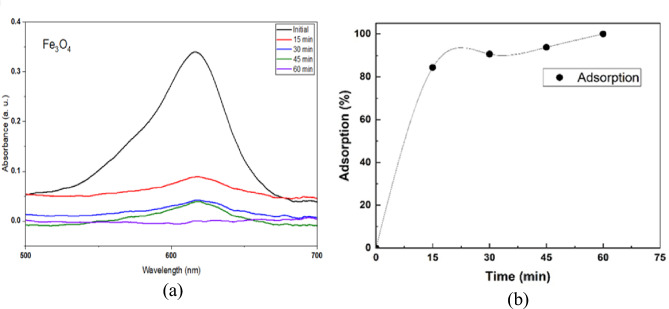
(**a**) The UV absorption spectrum of MG on the Fe_3_O_4_ NPs in the adsorption process, and (**b**) The adsorption activity of Fe_3_O_4_ NPs against the amount of MG dye.

The UV absorption spectrum and adsorption performance of Fe_3_O_4_ NPs against MG dye are illustrated in Fig. 9 (a, b). The results revealed that the Fe_3_O_4_ NPs exhibited significant adsorption effeciency ,  reaching 100% removal after 60 min. The high adsorption performance for the Fe_3_O_4_ NPs can be attributed to the high surface area^58^, which provides various active sites for dye adsorption . Furthermore, the presence of surface hydroxyl (-OH) groups enhances the interaction with the positively charged MG dye, consistence with the FTIR analysis ^59^.

#### Photocatalytic degradation activity

**Fig. 10:**
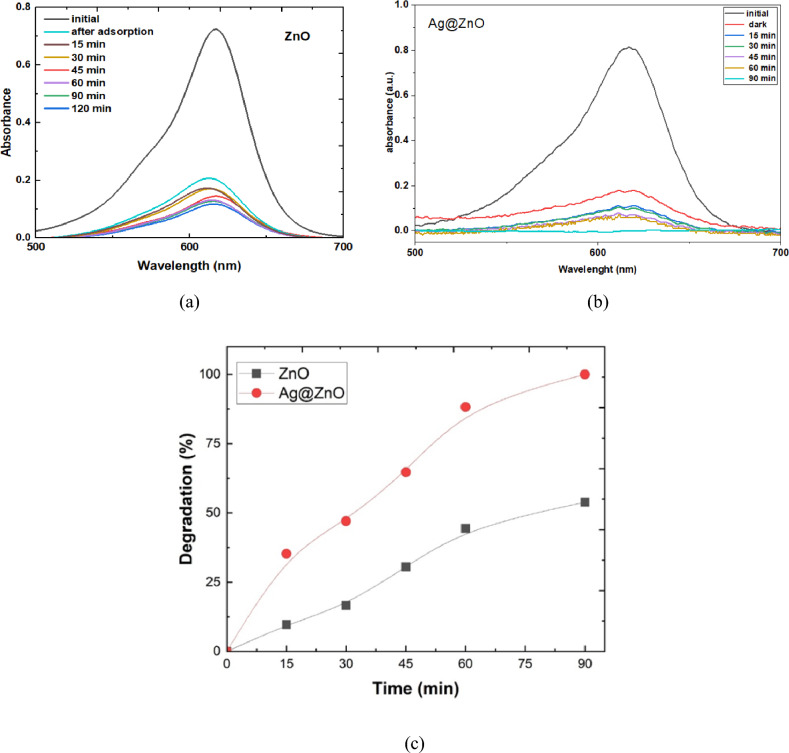
The UV–Vis absorbance of MG dye in the photocatalytic degradation process by (**a**) ZnO NRs, (**b**) Ag@ZnO nanocomposite, (**c**) The photocatalytic degradation activity of ZnO and Ag@ZnO nanocomposite.

The photocatalytic degradation of MG dye at an initial concentration of 10 ppm was evaluated using ZnO NRs and Ag@ZnO nanocomposite photocatalysts under visible-light irradiation, as illustrated in Fig. 10 (a, b).  The UV–Vis absorption spectra of MG dye recorded during the photocatalytic process over ZnO NRs and Ag@ZnO nanocomposites exhibit a progressive decline in the characteristic absorption peak, confirming effective degradation of the dye. The results revealed that the ZnO photocatalyst exhibited significant photocatalytic activity, achieving a degradation efficiency of 55% after 90 min of irradiation.  This finding demonstrates the effectiveness of ZnO as a photocatalyst for the degradation of MG dye under visible-light irradiation.

Furthermore, the Ag@ZnO nanocomposite demonstrated remarkable performance, achieving complete degradation within the same time frame. The enhanced photocatalytic activity of both ZnO and Ag@ZnO under visible-light irradiation can be attributed to the generation of electron–hole pairs upon absorption of photons. These photoexcited charge carriers participate in redox reactions with adsorbed oxygen and water molecules, leading to the formation of highly reactive species such as hydroxyl radicals (^•^OH) and superoxide radicals (^•^O_2_^−^). The generated ROS then reacts with the organic molecules of MG dye, breaking down its molecular structure and ultimately leading to its degradation into smaller, less harmful byproducts^60^.

The enhancement in photocatalytic activity observed with Ag@ZnO nanocomposite compared to pristine ZnO NRs can be attributed to the synergistic effects between Ag NPs and ZnO NRs, which promote the generation of reactive oxygen species (ROS) and improve the separation and utilization of photoinduced charge carriers^61^.

In addition to its catalytic activity, the presence of Ag NPs in the Ag@ZnO photocatalyst contributes to an increase in surface area and active sites, thereby enhancing the adsorption capacity towards organic pollutants such as MG dye^62^.

**Fig.11:**
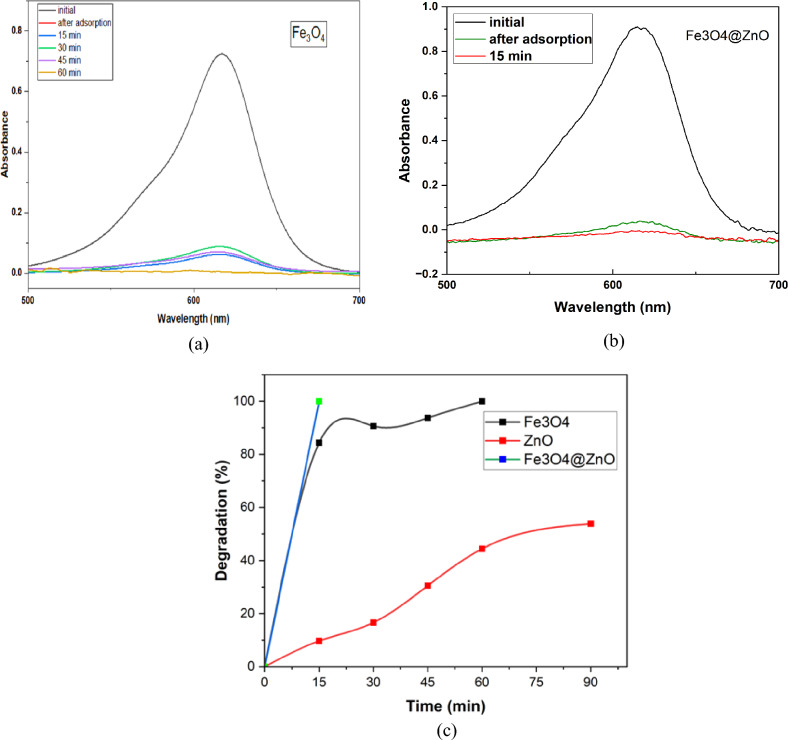
(**a**) The UV–Vis absorbance of MG with (**a**) Fe_3_O_4_ NPs, (**b**) Fe_3_O_4_ @ZnO nanocomposite, (**c**) the degradation activity of ZnO, Fe_3_O_4_, and Fe_3_O_4_@ZnO nanocomposite.

UV–Vis’s absorption spectra of MG dye solution before and after irradiation in the presence of Fe_3_O_4_ as a green magnetite NPs photocatalyst are shown in Fig. 11 (a). The photocatalytic degradation of the MC dye increased gradually with increasing irradiation time. After 60 min of light irradiation, the degradation activity reaches 100%.

The Fe_3_O_4_ NPS is used to enhance the photocatalytic degradation activity of the ZnO NRs, as illustrated in Fig. 11 (b). The results show that after 15 min,  the Fe₃O₄@ZnO nanocomposite achieved 100% photocatalytic degradation, whereas ZnO NRs exhibited only 10% degradation. The enhanced degradation activity of Fe_3_O_4_@ZnO nanocomposite is due to the high adsorption activity of magnetite NPs, as shown in Fig. 9 (b). In addition to the Fe_3_O_4_ NPs may decrease the e–h recombination rate and increase the light absorption may decrease the e–h recombination rate and increase the light absorption^63^.

#### The Ag@ZnO nanocomposite as a SERS substrate for MG dye sensing

**Fig. 12:**
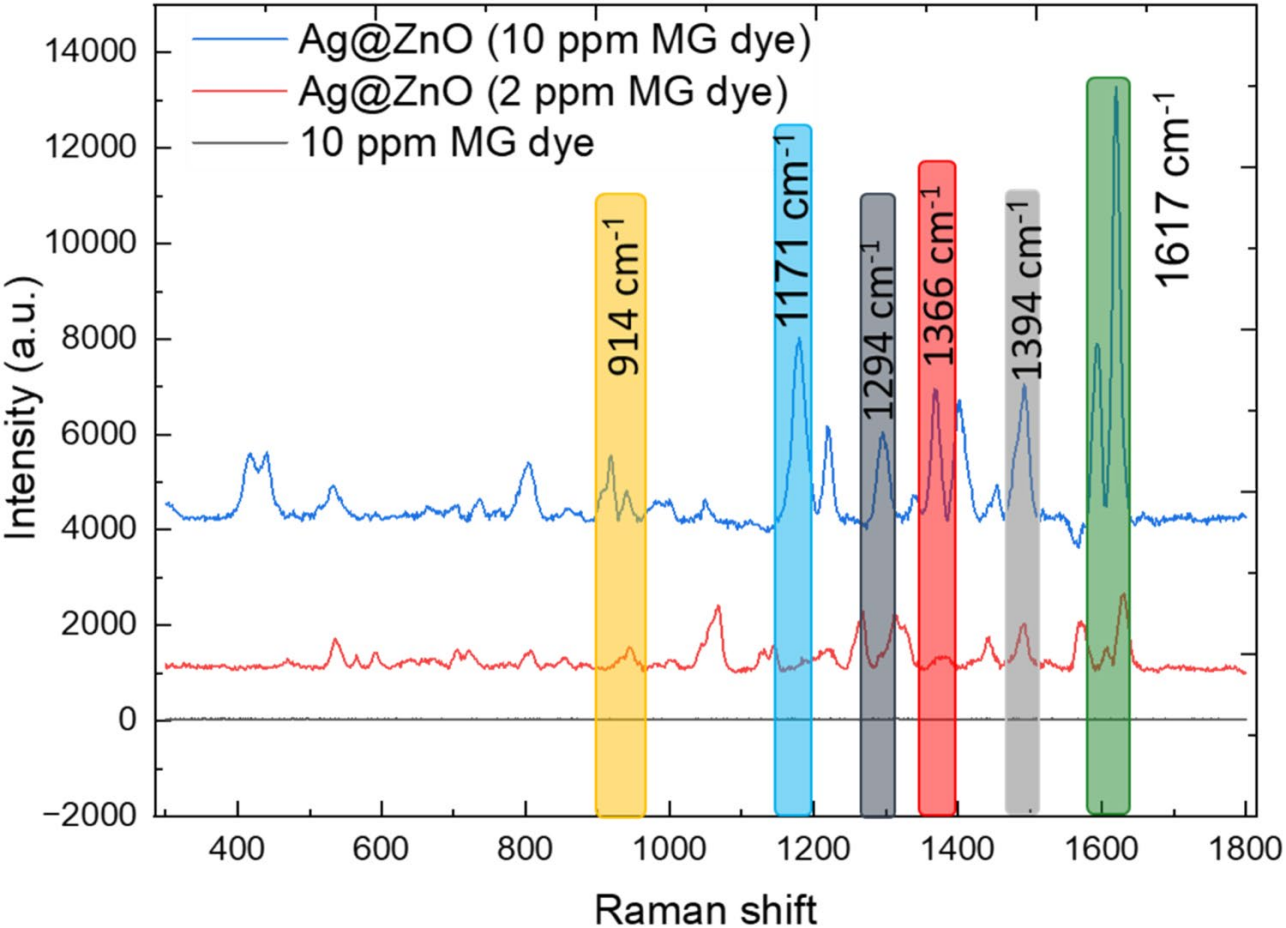
(**a**) Raman spectrum of malachite green (MG) at 20 ppm. (**b**) SERS spectrum of MG at 20 ppm adsorbed on the Ag@ZnO nanocomposite. (**c**) SERS spectrum of MG at 2 ppm on Ag@ZnO.

Surface-enhanced Raman spectroscopy (SERS) was employed to assess the sensing performance of Ag@ZnO nanocomposites toward MG dye (Fig. 12). As shown in the Raman spectra, MG dye at a concentration of 20 ppm exhibits no observable Raman signal when measured directly, reflecting the inherently weak Raman scattering of the dye under the applied experimental conditions. In contrast, for the same MG concentration deposited on the Ag@ZnO nanocomposite substrate, strong and well-defined Raman peaks are clearly detected, confirming a pronounced SERS effect. Furthermore, characteristic MG Raman bands remain clearly observable even at a much lower concentration of 2 ppm, demonstrating the high sensitivity of the Ag@ZnO substrate. The significant enhancement is mainly attributed to the localized surface plasmon resonance (LSPR) of Ag nanoparticles, which generates intense electromagnetic hot spots at the Ag–ZnO interface, thereby amplifying the Raman signal of MG molecules adsorbed on the surface. In addition, a chemical enhancement contribution arising from charge-transfer interactions between MG molecules and the Ag@ZnO nanocomposite further strengthens the Raman response. The prominent SERS peaks observed at approximately 914, 1171, 1294, 1366, 1394, and 1617 cm^−1^ are assigned to ring skeletal radial vibration, aromatic C–H in-plane bending, N–C bonding and C–C stretching, N–C stretching, C–C and C–H in-plane motions, and combined N–C bonding and C–C stretching vibrations, respectively^64^. These results clearly demonstrate that Ag@ZnO nanocomposite serve as effective and reliable SERS substrates for the sensitive detection of MG dye.

### Photocatalytic degradation mechanism

The band edge positions of ZnO were calculated using the electronegativity method according to the following Eqs. (11, 12):

11 $${\mathrm{E}}_{{{\mathrm{VB}}}} = {\mathrm{X}} - {\mathrm{E}}_{{\mathrm{e}}} + 1/2{\text{ E}}_{{\mathrm{g}}}$$

12 $${\mathrm{E}}_{{{\mathrm{CB}}}} = {\mathrm{E}}_{{{\mathrm{VB}}}} - {\mathrm{E}}_{{\mathrm{g}}}$$

where E_VB_ and E_CB_ represent the valence band and conduction band potentials of ZnO, expressed in electron volts versus the normal hydrogen electrode (eV vs. NHE), X is the absolute electronegativity of ZnO, E_e_ is the energy of free electrons on the hydrogen scale (4.5 eV), and E _g_ is the band gap energy. For the ZnO nanorods, the band gap was determined to be 3.08 eV, and the absolute electronegativity of ZnO was taken as 5.79 eV The energy of free electrons on the hydrogen scale was considered as 4.5 eV^65^.

Substituting these values into Eq. (11), the valence band potential was calculated as follows:

$${\mathrm{E}}_{{{\mathrm{VB}}}} = { 5}.{79} - {4}.{5} + {1}/{2}\left( {{3}.0{8}} \right)$$

$${\mathrm{E}}_{{{\mathrm{VB}}}} = + {2}.{83}\;{\mathrm{eV}}\;{\mathrm{vs}}.\;{\mathrm{NHE}}$$

The conduction band potential was then obtained using Eq. (12):

$${\mathrm{E}}_{{{\mathrm{CB}}}} = {2}.{83} - {3}.0{8}$$

$${\mathrm{E}}_{{{\mathrm{CB}}}} = - 0.{25}\;{\mathrm{eV}}\;{\mathrm{vs}}.\;{\mathrm{NHE}}$$

The Ag@ZnO nanocomposite is formed through the deposition of metallic silver (Ag) nanoparticles onto the surface of ZnO nanorods, resulting in the formation of a metal–semiconductor heterostructure. ZnO is an n-type semiconductor with a conduction band (CB) position of − 0.25 eV and a valence band (VB) position of + 2.83 eV versus the normal hydrogen electrode (NHE), corresponding to a band gap of 3.08 eV.

**Fig. 13:**
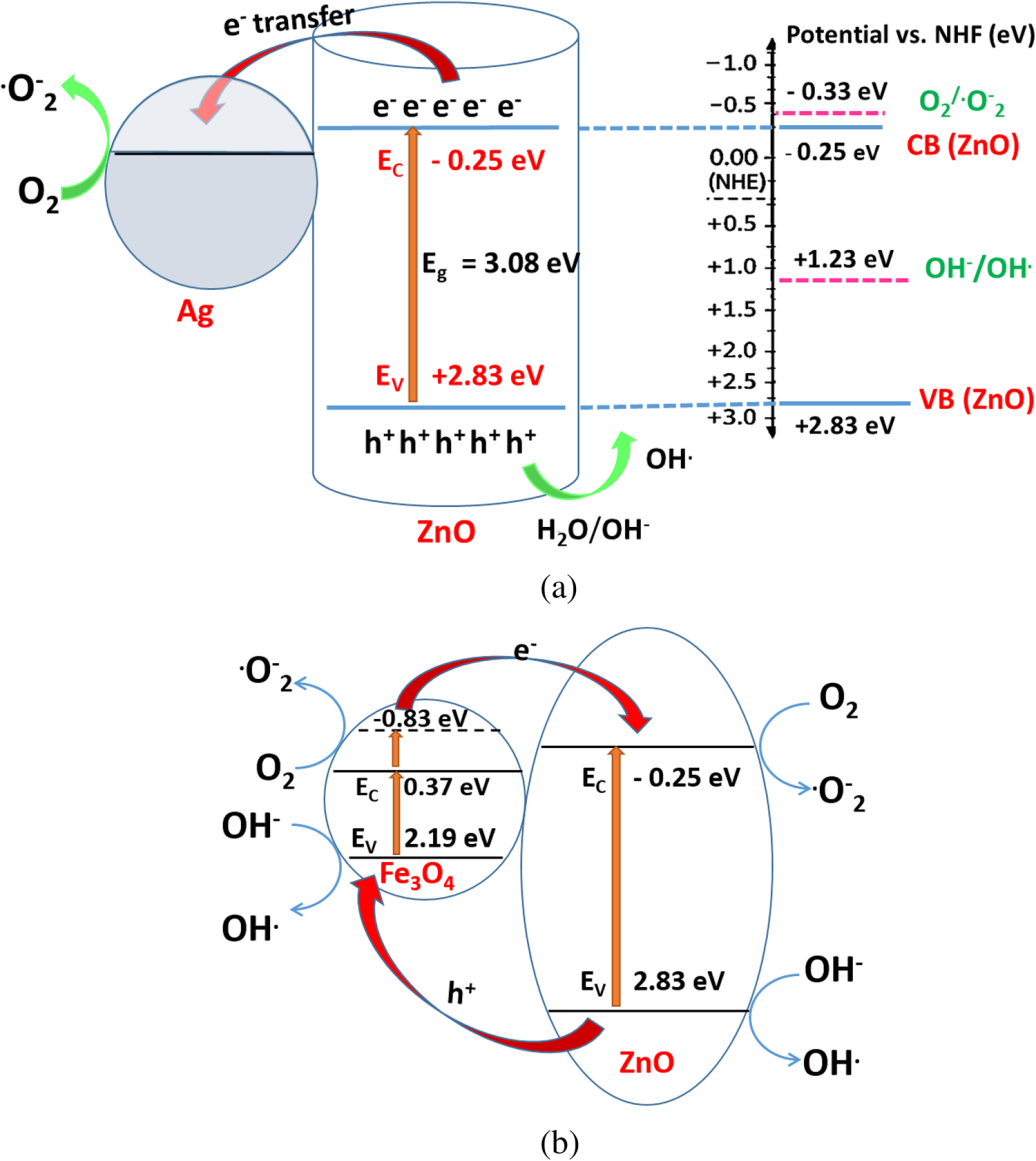
Schematic illustration of the photocatalytic degradation mechanism over (**a**) Ag@ZnO, and (**b**) ZnO@Fe_3_O_4_ nanocomposites.

When Ag nanoparticles are introduced, intimate interfacial contact is established between metallic Ag and ZnO. Due to the difference in Fermi levels between the metal and the semiconductor, electrons migrate from ZnO to Ag until thermodynamic equilibrium is achieved. This charge redistribution results in Fermi level alignment and the formation of a Schottky barrier at the Ag/ZnO interface as illustrated in Fig. 13. Ag nanoparticles act as electron sinks, capturing electrons from the conduction band of ZnO. This process enhances charge carrier separation and prolongs the lifetime of holes in the valence band. In addition, Ag nanoparticles exhibit surface plasmon resonance (SPR), which enhances visible-light absorption and may generate energetic electrons that contribute to improved photocatalytic activity.

Upon light irradiation (hν ≥ 3.08 eV), ZnO absorbs photons and generates electron–hole pairs (Eq. 13):

13 $${\mathrm{ZnO}} + {\mathrm{h}}\nu \to {\mathrm{e}}^{ - } \left( {{\mathrm{CB}}} \right) + {\mathrm{h}}^{ + } \left( {{\mathrm{VB}}} \right)$$

The photogenerated electrons accumulated on Ag nanoparticles are efficiently transferred to adsorbed oxygen molecules. Although the conduction band potential of ZnO (− 0.25 V vs. NHE) is slightly less negative than the redox potential of O_2_/^•^O_2_^−^ (− 0.33 V vs. NHE) (Eq. 14), the formation of superoxide radicals (^•^O_2_^−^) is still facilitated in the Ag@ZnO system. This is attributed to the synergistic effects of the heterojunction and plasmonic Ag nanoparticles. Ag acts as an electron sink and catalytic center for oxygen adsorption and activation, lowering the kinetic barrier for O_2_ reduction. Moreover, plasmon-induced energetic electrons from Ag under light irradiation further enhance the generation of ^•^O_2_^−^ radicals.

14 $${\mathrm{O}}_{2} + {\mathrm{e}}^{ - } \to {\mathrm{O}}_{2}^{ \bullet - }$$

The generated superoxide radical (O_2_^•−^) can further participate in reactions leading to the formation of reactive oxygen species (ROS), including hydroxyl radicals (^•^OH), which are highly oxidative and capable of degrading organic pollutants.

Because Ag collects photogenerated electrons from ZnO, the electron density available for oxygen reduction increases, leading to enhanced superoxide radical production.

The valence band potential (+ 2.83 eV vs. NHE) is sufficiently positive to oxidize water or hydroxide ions (Eq. 15):

15 $${\mathrm{H}}_{{2}} {\mathrm{O}}/{\mathrm{H}}^{ - } + {\mathrm{h}}^{ + } \to^{ \bullet } {\mathrm{OH}} + {\mathrm{H}}^{ + }$$

The generated hydroxyl radicals (^•^OH) are strong oxidizing agents that attack dye molecules, causing chromophore cleavage, ring-opening reactions, and eventual mineralization into CO_2_ and H_2_O.

As calculated previously, the conduction band (CB) and valence band (VB) potentials of ZnO are − 0.25 eV and + 2.83 eV (vs NHE), respectively, whereas Fe_3_O_4_ exhibits band edge positions of approximately + 0.37 eV for the CB and + 2.19 eV for the VB. This band alignment allows the formation of an interfacial heterojunction that promotes effective charge separation between the two semiconductors.

Upon irradiation with photons (of energy around 3 eV), Fe_3_O_4_, which possesses a relatively narrow bandgap, is readily photoexcited. Electrons in the top of VB of Fe_3_O_4_ are promoted to higher excited states above its conduction band, reaching an energy level of approximately − 0.83 eV (vs NHE), as illustrated in Fig. 13 b. Because this excited level is more negative than the CB potential of ZnO (− 0.25 eV), the photogenerated electrons possess sufficient driving force to transfer from Fe_3_O_4_ to the CB of ZnO across the heterojunction interface. These electrons subsequently react with dissolved oxygen molecules to produce superoxide radicals (^•^O_2_^−^), which contribute to the degradation process.

Meanwhile, the photogenerated holes migrate in the opposite direction and accumulate in the VB of Fe_3_O_4_, where they can oxidize surface hydroxide ions or water molecules to form hydroxyl radicals (^•^OH). The spatial separation of electrons on the ZnO side and holes on the Fe_3_O_4_ side effectively suppresses electron–hole recombination and enhances the generation of reactive oxygen species such as ^•^O_2_^−^ and ^•^OH. These highly reactive radicals subsequently attack and oxidatively decompose Malachite Green molecules, leading to their degradation and eventual mineralization. Consequently, the synergistic interaction between ZnO and Fe_3_O_4_ significantly enhances the photocatalytic performance of the composite system.

## Conclusion

In this study, ZnO NRs, Ag@ZnO, and Fe_3_O_4_@ZnO nanocomposites were successfully synthesized using a microwave-assisted hydrothermal approach and systematically investigated for wastewater remediation, while Ag@ZnO nanocomposite was employed as the substrate for dye sensing applications. Structural and spectroscopic analyses confirmed the formation of highly crystalline ZnO NRs, efficient decoration with plasmonic Ag nanoparticles, and successful integration of magnetic Fe_3_O_4_ NPs. Optical characterization revealed enhanced visible-light absorption and reduced electron–hole recombination in Ag@ZnO, while Fe_3_O_4_ contributed strong adsorption capacity and broad optical absorption. Photocatalytic experiments demonstrated that Ag@ZnO and Fe_3_O_4_@ZnO nanocomposites achieved complete degradation of MG dye under visible-light irradiation, outperforming pristine ZnO due to improved charge separation, increased active sites, and synergistic interfacial effects. Moreover, Ag@ZnO exhibited excellent SERS performance, enabling sensitive detection of MG down to 2 ppm through localized surface plasmon resonance and chemical enhancement mechanisms. These findings highlight the potential of plasmonic and magnetic ZnO-based nanocomposites as multifunctional platforms for efficient dye degradation and ultrasensitive pollutant detection, offering a promising strategy for sustainable wastewater treatment and environmental monitoring. The comparative study of Ag@ZnO and Fe_3_O_4_@ZnO nanocomposites demonstrates how plasmonic Ag and magnetic Fe_3_O_4_ distinctly modify ZnO activity, enabling enhanced photocatalysis and SERS sensing in the former and improved adsorption-assisted photocatalysis and recyclability in the latter. Improved photocatalytic activity arises from effective charge separation in Ag@ZnO and Fe_3_O_4_@ZnO nanocomposites, leading to enhanced ROS-driven degradation.

## Data Availability

The datasets used and/or analysed during the current study available from the corresponding author on reasonable request.
